# A Framework to Support Automated Classification and Labeling of Brain Electromagnetic Patterns

**DOI:** 10.1155/2007/14567

**Published:** 2007-12-06

**Authors:** Gwen A. Frishkoff, Robert M. Frank, Jiawei Rong, Dejing Dou, Joseph Dien, Laura K. Halderman

**Affiliations:** ^1^Learning Research and Development Center, University of Pittsburgh, Pittsburgh, PA 15260, USA; ^2^NeuroInformatics Center, University of Oregon, 1600 Millrace Drive, Eugene, OR 97403, USA; ^3^Computer and Information Sciences, University of Oregon, Eugene, OR 97403, USA; ^4^Department of Psychology, University of Kansas, 1415 Jayhawk Boulevard, Lawrence, KS 66045, USA

## Abstract

This paper describes a framework for automated classification and labeling of patterns in electroencephalographic (EEG) and magnetoencephalographic (MEG) data. We describe recent progress on four goals: 1) specification of rules and concepts that capture expert knowledge of event-related potentials (ERP) patterns in visual word recognition; 2) implementation of rules in an automated data processing and labeling stream; 3) data mining techniques that lead to refinement of rules; and 4) iterative steps towards system evaluation and optimization. This process combines top-down, or knowledge-driven, methods with bottom-up, or data-driven, methods. As illustrated here, these methods are complementary and can lead to development of tools for pattern classification and labeling that are robust and conceptually transparent to researchers. The present application focuses on patterns in averaged EEG (ERP) data. We also describe efforts to extend our methods to represent patterns in MEG data, as well as EM patterns in source (anatomical) space. The broader aim of this work is to design an ontology-based system to support cross-laboratory, cross-paradigm, and cross-modal integration of brain functional data. Tools developed for this project are implemented in MATLAB and are freely available on request.

## 1. INTRODUCTION

The complexity of brain electromagnetic (EM) data has led to a variety of processes for EM pattern classification and labeling over the past several decades. The absence of a common framework may account for the dearth of statistical metaanalyses in this field. Such cross-lab, cross-paradigm reviews are critical for establishing basic findings in science. However, reviews in the EM literature tend to be informal, rather than statistical: it is difficult to generalize across datasets that are classified and labeled in different ways.

To address this problem, we have designed a framework to support automated classification and labeling of patterns in electroencephalographic (EEG) and magnetoencephalographic (MEG) data. In the present paper, we describe the framework architecture and present an application to averaged EEG (event-related potentials, or ERP) data collected in a visual word recognition paradigm. Results from this study illustrate the importance of combining top-down and bottom-up approaches. In addition, they suggest the need for ongoing system evaluation to diagnose potential sources of error in component analysis, classification, and labeling. We conclude by discussing alternative analysis pathways and ways to improve efficiency of implementation and testing of alternative methods. It is our hope that this framework can support increased collaboration and integration of ERP results across laboratories and across study paradigms.

### 1.1. Classification of ERPs

A standard technique for analysis of EEG data involves averaging across segments of data (*trials*), time-locking to stimulus or response *events*. The resulting measures are characterized by a sequence of positive and negative deflections distributed across time and space (scalp locations). In principle, activity that is not event-related will tend towards zero as the number of averaged trials increases. In this way, ERPs provide increased signal-to-noise, and thus increased sensitivity to functional, (e.g., task) manipulations. Signal averaging assumes that the brain signals of interest are time-locked to (or “evoked by”) the events of interest. As illustrated in recent work on induced (nontime-locked) versus evoked (time-locked) EEG activity, this assumption does not always hold ([1, 2]).

In the past several decades, researchers have described several dozen spatiotemporal ERP patterns (or *components*), which are thought to index a variety of neuropsychological processes. Some patterns are observed across a range of experimental contexts, reflecting domain-general processes, such as memory, decision-making, and attention. Other patterns are observed in response to specific types of stimuli, reflecting human expertise in domains such as mathematics, face recognition, and reading comprehension (for reviews see [3, 4]). Previous investigations of these patterns have demonstrated the effectiveness of ERP methods for addressing basic questions in nearly every area of psychology.

Given the success of this methodology, ERPs are likely to remain at the forefront of research in clinical and cognitive neuroscience, even as newer methods for EEG and MEG analyses are developed as alternatives to signal averaging (e.g., [1, 2, 5–7]).

**Figure 1:**
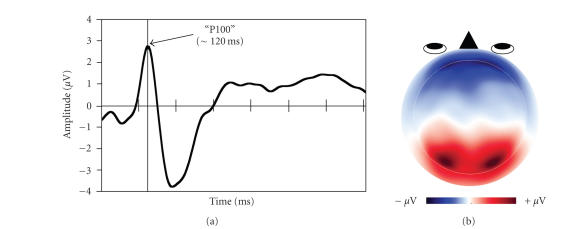
(a) Time course of P100 pattern, plotted at left occipital electrode, O1. Time is plotted on the x-axis (0–700 milliseconds); each vertical hash mark represents 100 milliseconds. Amplitude is plotted on the y-axis (scale, ±4 μV). The dark vertical line marks the time of peak amplitude (∼120 milliseconds). (b) Scalp topography of the P100 pattern, plotted at the time of peak amplitude. Red, positive. Blue, negative.

At the same time, ERP methods face some important challenges. A key challenge is to identify standardized methods for measure generation, as well as objective and reliable methods for identification and labeling of ERP components. Traditionally, researchers have characterized ERP components in respect to both physiological (spatial, temporal) and functional criteria [8, 9]. Physiological criteria include latency and scalp distribution, or topography. For example, as illustrated in Figure 1, the visual “P100 component” is characterized by a positive deflection that peaks at ∼100 milliseconds after onset of a visual stimulus (A) and is maximal over occipital electrodes, reflecting activity in visual cortex (B).

Despite general agreement on criteria for ERP component identification [9], in practice such patterns can be hard to identify, particularly in individual subjects. This difficulty is due in part to the superposition of patterns generated by multiple brain regions at each time point [10], leading to complex spatial patterns that reflect the mixing of underlying patterns. Given this complexity, ERP researchers have adopted a variety of solutions for scalp topographic analysis (e.g., [11, 12]). It can therefore be difficult to compare results from different studies, even when the same experimental stimuli and task are used.

Similarly, researchers use a variety of methods for describing temporal patterns in ERP [13]. For example, early components, such as the P100, tend to be characterized by their peak latency, while the time course of later components, such as the N400 or P300, is typically captured by averaging over time “windows” (e.g., 300–500 milliseconds). The latency of other components, such as the N400, has been quantified in a variety of ways. Finally, there is variability in how functional information (e.g., subject-, stimulus-, or task-specific variables) is used in ERP pattern classification. Some patterns, such as the P100, are easily observed as large deflections in the raw ERP waveforms. Other patterns, such as the mismatch negativity are more reliably seen in difference measures, calculated by subtracting ERP amplitude in one condition from the ERP amplitude in a contrasting condition. This inconsistency may lead to confusion, particularly when the same label is used to refer to two different measures, as is often the case.

### 1.2. Outline of paper

In summary, the complexity of ERP data has led to multiple processes for measure generation and pattern classification that can vary considerably across different experiment paradigms and across research laboratories. Ultimately, this limits the ability both to replicate prior results and to generalize across findings to achieve high-level interpretations of ERP patterns.

In light of these challenges, the goal of this paper is to describe a framework for automated classification and labeling of ERP patterns. The framework presented here comprises both *top-down* (knowledge-driven) and *bottom-up* (data-driven) methods for ERP pattern analysis, classification, and labeling. Following, we describe this framework in detail in Section 2 and present an application to patterns in ERP data from a visual word processing paradigm in Section 3. Section 4 describes approaches to system evaluation. Section 5 describes data mining for refinement of expert-driven (top-down) methods. In Section 6, we draw some general conclusions and discuss extensions of our framework for representation of patterns in source space, and ontology development to support cross-paradigm, cross-laboratory, and cross-modal integration of results in EM research.

## 2. PATTERN CLASSIFICATION FRAMEWORK

**Figure 2:**
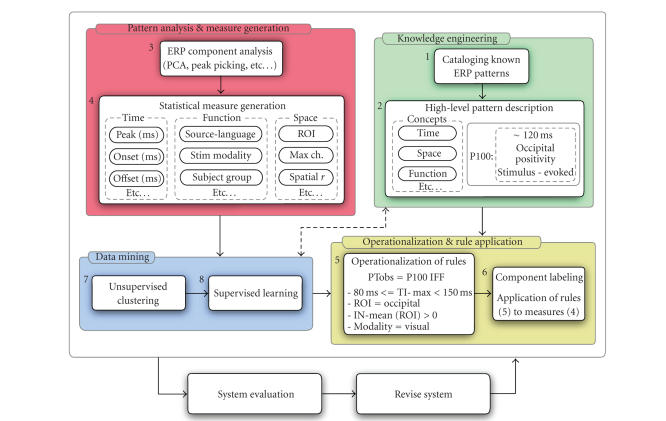
Pattern classification and labeling scheme. *Knowledge engineering* (processes 1, 2) includes “top-down” specification of ERP concepts and rules, formulated by domain experts. *Component analysis* and *measure generation* (processes 3, 4) yield summary metrics that are used for pattern classification and labeling. Implementation and operationalization of pattern rules (processes 5, 6) are detailed in Section 2. *Data mining* (processes 7, 8) includes “bottom-up” or data-driven methods for clustering and discovery of pattern rules (Section 5). *System evaluation* is detailed in Section 4.

As illustrated in Figure 2, our framework comprises five main processes.

- *Knowledge engineering.* Known ERP patterns are cataloged (1). High-level rules and concepts are described for each pattern (2).
- *Pattern analysis * and *measure generation*. Analysis methods are selected and applied to ERP data (3). The goal is transformation of continuous spatiotemporal data into discrete patterns for labeling. Statistics are generated (4) to capture the rules and concepts identified in (2).
- *Data mining*. Unsupervised clustering (7) and supervised learning (8) are used to explore how measures cluster, and how these clusters may be used to identify and label patterns using rules derived independently of expert knowledge.
- *Operationalization and application of rules*. Rules are operationalized by combining metrics in (4) with prior knowledge (2). Data mining results (7-8) may be used to validate and refine the rules. Rules are applied to data, using an automated labeling process (6) detailed below.

Following we describe how these processes have been implemented in a series of MATLAB procedures. We then report results from the application of this process to data from a visual word processing experiment. Results are evaluated against a “gold standard” that consists of expert judgments regarding the presence or absence of patterns, and their prototypicality, for each of 144 observations (36 subjects ×4 experiment conditions).

### 2.1. Knowledge engineering (process 1, 2)

The goal of knowledge engineering is to identify concepts that have been documented for a particular research domain. Based on prior research on visual word processing we have tentatively identified eight spatiotemporal patterns that are commonly observed from ∼100 to ∼700 milliseconds after presentation of a visual word stimulus, including the P100, N100, late N1/N2b, N3, P1r, MFN, N400, and P300. Space limitations preclude a detailed discussion of each pattern (see reviews in [3, 4]). The left temporal N3 and medial frontal negativity (MFN) components are less well known, but have been described in several high-density ERP studies of visual word processing (e.g., [14–16]). The P1r [17] has also been referred to as a posterior P2 [18]. The late N1/N2b has variously been referred to as an N2, an N170, and a recognition potential (see [15] for discussion and references). It is not clear that the late N1/N2 represents a component that is functionally distinct from the N1 and N3, though it sometimes emerges in tPCA results as a distinct spatiotemporal pattern (e.g., see Section 3). These eight patterns reflect a working taxonomy of ERP in research on visual word processing between ∼60–700 milliseconds. Application of the present framework to large numbers of datasets collected across a range of paradigms, and across different ERP research labs, would contribute to the refinement of this taxonomy.

A note of caution is in order, concerning the labels for scalp regions of interest (ROIs). By convention, areas of the scalp are associated with anatomical labels, such as “occipital,” “parietal,” “temporal,” and “frontal” (see Table 1). It is well known, however, that a positive or negative deflection over a particular scalp ROI is not necessarily generated in cortex directly below the measrured data. ERP patterns can reflect sources tangential to the scalp surface. In this instance, the positive and negative fields may be maximal over remote regions of the scalp, reflecting a dipolar scalp distribution (e.g., with a positive maximum over frontal scalp regions, and a negative maximum over temporal scalp regions). Thus, the ROI labels should not be interpreted as literal references to brain regions. The ROI clusters used in the present study are shown in Appendix A.

### 2.2. Data summary

Prior to analysis, ERP data consist of complex waveforms (time series), measured at multiple electrode sites. To simplify analysis and interpretation of these data, a standard practice is to transform the ERPs into discrete patterns. Traditional methods for data summary include identification of peak latency within a specified time window (“peak picking”) and computing the mean amplitude over a time window for each electrode (“windowed analysis”), or averaged over electrode clusters (regions of interest—*ROIs*). An alternative method is principal components analysis (PCA), which decomposes the data into “latent” patterns, or *factors*. The following subsection describes this method in detail, and explains the utility of PCA for automated pattern classification.

#### 2.2.1. Temporal PCA methods (process 3)

PCA belongs to a class of factor-analytic procedures, which use eigenvalue decomposition to extract linear combinations of variables (latent “factors”) in such a way as to account for patterns of covariance in the data parsimoniously, that is, with the fewest factors. Mathematically, the goal of PCA is to take intercorrelated variables ($x_{1},\ldots ,x_{n}$) and combine them such that the tranformed data, the “principal components” (PC), are linear combinations of x, weighted to maximize the amount of variance captured by each eigenvector ($v_{i}$):

(1) $${\text{PC}}_{1}=v_{11}x_{1}+v_{12}x_{2}+\cdots +v_{1n}x_{n}.$$

In this way, the original set of variables ($x_{1},\ldots ,x_{n}$) is “projected” into a new data space, where the dimensions of this new space are captured by a small number of latent factors (the eigenvectors).

In ERP data, the variables ($x_{1},\ldots ,x_{n}$) are the microvolt readings either at consecutive time points (temporal PCA) or at each electrode (spatial PCA). The major source of covariance isassumed to be the ERP components, characteristic features of the wave form that are spread across multiple time points and multiple electrodes. Ideally, each latent factor corresponds to a separate ERP component, providing a statistical decomposition of the brain electrical patterns that are superposed in the scalp-recorded data. To achieve this ideal factor-to-pattern mapping, the factors may be “rotated” so that the variance associated with the original variables (timepoints) is redistributed across the factors in such a way that maximizes “simple structure,” that is, that achieves a simple and transparent mapping from variables to factors. (See [19] for a review of PCA and related factor-analytic methods for ERP data decomposition.)

In the present application, we used temporal PCA (tPCA) as implemented in the Dien PCA Toolbox [20]. In temporal PCA, the data are organized with the variables corresponding to time points and observations corresponding to the different waveforms in the dataset. The waveforms vary across subjects, electrodes, and experimental conditions. Thus, subject, spatial, and task variance are collectively responsible for covariance among the temporal variables. The data matrix is then self-multiplied and mean-corrected to produce a covariance matrix. The covariance matrix is subjected to eigenvalue decomposition, and the resulting nonnoise factors are rotated using Promax to obtain a more transparent relationship between the PCA factors and the latent variables of interest (i.e., ERP components).

**Figure 3:**
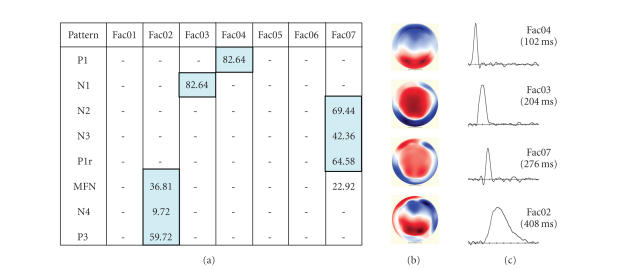
Autoclassification and labeling results. (a) Percentage of observations matching rule criteria for each pattern. (b) Topogragraphy and (c) time course of pattern factors.

After transformation of the ERP data into factor space, the data are projected back into the original data space, by multiplying factor scores by factor loadings and by the standard deviation at each timepoint (see the appendix in [21]). In this way, it is possible to visualize and extract information about the strength of the pattern at each electrode, to determine the spatial distribution of the pattern for a given subject and experiment condition. Visualizing the spatial projection of each factor in this way is useful in interpreting tPCA results (e.g., see Figure 3(b)).

For our initial attempts to automate data description and classification, tPCA offered several advantages over traditional methods. First, tPCA is able to separate overlapping spatiotemporal patterns. Second, tPCA automatically extracts a discrete set of temporal patterns. Third, when implemented and graphed appropriately, tPCA results are easily interpreted with respect to previous findings, as illustrated below. tPCA is therefore easily incorporated in an automated process for ERP pattern extraction and classification. In the final section, we address some limitations of tPCA as a method of ERP pattern analysis.

#### 2.2.2. Measure generation (process 4)

For each tPCA factor, we extracted 32 summary metrics that characterize spatial, temporal, and functional dimensions of the data. The full set of metrics, along with their definitions, is listed in Appendix C. Note that our expert-defined rules, which were used for the tPCA autolabeling process, mainly involved two metrics (see Section 2.2.3 for details): *In-mean (ROI)* and *TI-max*. In-mean (ROI) represents the amplitude over a region-of-interest (ROI), averaged over electrode clusters for each latent factor at the time of peak latency, after the factor has been projected back into channel space. TI-max is the peak latency and is measured on the factor loadings, which are sign-invariant.

Although these two metrics intuitively capture the spatial and temporal dimensions of the ERP data that are most salient to ERP researchers, our prior data mining results suggested that additional metrics might improve the tPCA autolabeling results [22, 23]. In particular, some failures in the autolabeling process (i.e., cases where the modal factor for a given pattern did not show a match to the rule in a given condition, for a given subject) were due to component overlap that remained even after tPCA. For example, in one of our four pilot datasets [23], the P100 pattern was partially captured by a factor corresponding to the N100. For some subjects, most of the P100 was in fact captured by this “N100 factor.” The factor showed a slow negativity, beginning before the stimulus onset, and the P100 appeared as a positive going deflection that was superposed on this sustained negativity. However, because the rule specified that the mean amplitude over the occipital electrodes should be positive, the factor did not meet the P100 rule criteria.

To address this issue, we implemented *onset and offset metrics*. Each onset latency was estimated as the midpoint of four consecutive sliding windows in which corresponding t-tests (threshold, P=.05) indicated that the means of their respective windowed signals diverged significantly from a baseline value, typically zero. The subsequent offset was the temporal midpoint at which the four consecutive t-tests showed their windowed signal means returned to baseline. The procedure is implemented as described in [24].

Using the onset latency to determine a “baseline” (0-point or onset) for each pattern, we then computed *peak-to-baseline* and *baseline-to-peak* metrics to capture phasic deflections that could be confused with slow potentials. The baseline intensity was computed as the signal mean within an interval centered on component onset. We predicted that data mining results would incorporate these measures to yield improved accuracy in the labeling process.

In addition, we added metrics to capture variations in amplitude due to experimental variables. Four measures were computed: *Pseudo-Known* (difference in response to nonwords versus words), *RareMisses-RareHits* (difference in response to unknown rare words versus words that we correctly recognized), *RareHits-Known* (difference in response to rare versus low-frequency words), and *Pseudo-RareMisses* (difference in nonwords versus missed rare words). Because prior research has shown that semantic processing can affect the N2, N3, MFN, N4, and P3 patterns, we predicted that the data mining procedures would identify one or more of these metrics as important for pattern classification.

#### 2.2.3. Rule operationalization (process 5)

Rules for each ERP pattern were formulated initially based on results from prior literature and were operationalized using metrics defined in Process 4 (Section 2.2.2). After application of the initial rules to test data, we evaluated the results against a “Gold Standard” (see Section 4 for details) and modified the pattern rules to improve accuracy. For example, after initial testing, the visual “P100” pattern (P100v) was defined as follows: for any n, ${\text{FA}}_{n}=$ P100v *if and only if*

- 80 ms < TI-max (FA_n_) ≤150 milliseconds,
- In-mean(ROI) >0,
- EVENT (FA_n_) = stimon,
- MODALITY (EVENT) = visual,

where FA_n_ is defined as the nth tPCA factor, and P100v is the visual-evoked P100 (“v” stands for “visual”). TI-max is the time of peak amplitude, In-mean(ROI) is the mean amplitude over the region-of-interest (ROI), and ROI for P100v is specified as “occipital” (i.e., mean intensity over occipital electrodes). “Stimon” refers to stimulus onset, which is the event that is used for time-locking single trials to derive the ERP. “MODALITY” refers to the stimulus modality(e.g., visual, auditory, somatosensory, etc.). See Appendix B for a full listing of rule formulae. 

 These rules represent informed hypotheses, based on expert knowledge. As described below (Section 5), bottom-up methods can be used to refine these rules. Further, as the rules are applied to larger and more diverse sets of data, they are likely to undergo additional refinements (see Section 4.1).

#### 2.2.4. Automated labeling (process 6)

 For each condition, subject, and tPCA factor, we used MATLAB to compute temporal and spatial metrics on that factor's contribution to the scalp ERP. The values of the metrics specified in the expert defined rules were then compared to rule-specific thresholds that characterized specific ERP components. Thresholds were determined through expert definitions that were formulated and tested as described in Section 2.2.3). The results of the comparisons were recorded in a true/false table, and factors meeting all criteria were flagged as capturing the specified ERP component for that subject and condition. All data were automatically saved to Excel spreadsheets organized by rule, condition, and subject. 

### 2.3. Data mining

 As described in Section 2.1, ERP patterns are typically discovered through a “manual” process that involves visual inspection of spatiotemporal patterns and statistical analysis to determine how the patterns differ across experiment conditions. While this method can lead to consensus on the high-level rules and concepts that characterize ERP patterns in a given domain, operationalization of these rules and concepts is highly variable across research labs, as described in Section 1. Bottom-up (data-driven) methods can contribute to standardization of rules for classifying known patterns, and possibly to discovery of new patterns, as well. Here we describe two bottom-up methods, unsupervised learning (i.e., clustering) and supervised learning (i.e., decision tree classifiers). 

#### 2.3.1. Clustering (process 7)

 In this study, we used the expectation-maximization (EM) algorithm for clustering [25], as implemented in WEKA [26]. EM is used to approximate distributions using mixture models. It is an procedure that iterates around the expectation (E) and maximization (M) steps. In the E-step for clustering, the algorithm calculates the posterior probability, $h_{ij}$, that a sample j belongs to a cluster $C_{i}$:

(2) $$h_{ij}=P(C_{i}|D_{j})=\frac{p(D_{j}|\theta_{i})\pi_{i}}{\sum_{m=1}^{C}p(D_{j}|\theta_{m})\pi_{m}},$$

where $\pi_{i}$ is the weight for the ith mixture component, $D_{j}$ is the measurement, and $\theta_{i}$ is the set of parameters for each density functions. In the M-step, the EM algorithm searches for optimal parameters that maximize the sum of weighted log-likelihood probabilities. EM automatically selects the number of clusters by maximizing the logarithm of the likelihood of future data. Observations that belong to the same pattern type should ideally be assigned to a single cluster.

#### 2.3.2. Classification (process 8)

We use a traditional classification technique, called a decision tree learner. Each internal node of a decision tree represents an attribute, and each leaf node represents a class label. We used J48 in WEKA, which is an implementation of C4.5 algorithm [27]. The input to the decision tree learner for the present study consisted of a pattern factor metrics vector of dimension 32, representing the 32 statistical metrics (Appendix C). Cluster labels were used as classification labels. The labeled data set was recursively partitioned into small subsets as the tree was being built. If the data instances in the same subset were assigned to the same label (class), the tree building process was terminated. We then derived If-Then rules from the resulting decision tree and compared them with expert-generated rules.

## 3. APPLICATION: VISUAL WORD PROCESSING

The ERP data for this study consisted of 144 observations (36 subjects ×4 experiment conditions) that were acquired in a lexical decision task (see [28] for details). Participants viewed word and pseudoword stimuli that were presented, one stimulus at a time, in the center of a computer monitor and made word/nonword judgments to each stimulus using their right index and middle fingers to depress the “1” and “2” keys on a keyboard (“yes” key counterbalanced across subjects). Stimuli consisted of 350 words and word-like stimuli, including low-frequency words that were familiar to subjects (based on pretesting) and rare words like “nutant” (and unlikely to be known by participants). Letters were lower-case Geneva black, 26 dpi, presented foveally on a white screen. Words and nonwords were matched in mean length and orthographic neighborhood [29, 30].

### 3.1. ERP experiment data

ERP data were recorded using a 128-channel electrode array, with vertex recording reference [31]. Data were sampled at a rate of 250 per second and were amplified with a 0.01 Hz highpass filter (time constant ∼10 seconds). The raw EEG was segmented into 1500 milliseconds epochs, starting 500 milliseconds before onset of the target word. There were four conditions of interest: correctly classified, low-frequency words (*Known*); correctly classified rare words (*RareHits*), rare words rated as nonwords (*RareMisses*); and correctly classified nonwords (*Pseudo*).

Segments were marked as bad if they contained ocular artifacts (EOG >70 μV), or if more than 20% of channels were bad on a given trial. The artifact-contaminated trials were excluded from further analysis.

Segmented data were averaged across trials (within subjects and within conditions) and digitally filtered with a 30-Hz lowpass filter. After further channel and subject exclusion, bad (excluded) channels were interpolated. The data re-referenced to the average of the recording sites [32], using a polar average reference to correct for denser sampling over superior, as compared with inferior, scalp locations [33, 34]. Data were averaged across individual subjects, and the resulting “grand-averaged” ERPs were used for inspection of waveforms and topographic plots.

## 4. TPCA AUTOLABELING RESULTS

Temporal PCA (tPCA) was used to transform the ERP data into a set of latent temporal patterns (see Section 2.2.1 for details). We extracted the first 15 latent factors from each of the four datasets, accounting for approximately 80% of the total variance.These 15 tPCA factors were then subjected to a Promax rotation.

After the tPCA factors were projected back into the original data space (Section 2.2.1), we applied our expert-defined rules to determine the percentage of observations that matched each target pattern. Results are shown in Table 2.

We assigned labels to the first 10 factors based on the correspondence between the target patterns and the tPCA factors. Results were as follows: Factor 4 = P100, Factor 3 = N100, Factor = N2, Factor 7 = N3/P1r, Factor 2 = MFN/N4, and Factor 9 = P3. Figure 3 displays the time course and topography for these six *pattern factors*.

Note that many patterns showed splitting across two or more factors. This may reflect misallocation of pattern variance across the factors (i.e., inaccuracies in the tPCA decomposition), inaccuracies in rule definitions, or both. A complementary problem is seen in the case of factors 2, 7, and 10, which show matches to more than one target pattern. Again, this may reflect misallocation of variance. Alternatively, these results may suggest a need to refine our pattern descriptions, the rules that are used to identify pattern instances, or both. In either case, these findings point to the need for systematic evaluation of results. Diagnosing potential sources of error is the first step towards systematic improvements of methods.

### 4.1. Evaluation of top-down methods

In our framework, top-down methods for pattern classification are dependent on the accuracy of both the data summary methods and the expert-defined rules. In particular,

1. data summary methods should yield discrete patterns that reflect different underlying neuropsychological processes, or “components;”
2. rules that are applied to summary metrics should be implemented in a way that effectively discriminates between separate patterns.

Our initial efforts have led to encouraging classification results, as illustrated above. However, several findings suggest the need to consider possible misallocation of variance in the data summary process, and ways of optimizing pattern rules.

#### 4.1.1. Diagnosing misallocation of variance

A well-known critique of PCA methods, including temporal PCA, is that inaccuracies in the decomposition can lead to misallocation of variance ([21, 35]). For example, in our results, the left temporal N3 and parietal P1r patterns were both assigned to a single factor (cf. [15] for similar results). Recent methods can achieve separation of patterns that have been confounded in an initial PCA (see [19] for a discussion). A more serious problem is that of the pattern splitting: well-known patterns like the P100 are expected to map to a single rule (factor). Indeed, this simple mapping was obtained in 3 or our 4 pilot datasets [23]. Splitting of the P100 across two factors therefore suggests a possible misallocation of variance in the tPCA. A future challenge will be to develop rigorous methods of diagnosing misallocation of variance in the decomposition of ERPs. In the final section, we consider alternatives to tPCA, which may address this issue.

#### 4.1.2. Comparison with a “gold standard”

The validity of our tPCA autolabeling procedures was assessed by comparing autolabeling results with a “gold standard,” which was developed through manual labeling of patterns. Two ERP analysts visually inspected the raw ERPs for each subject and each condition. For each target pattern, the analysts indicated whether the pattern was present, based on inspection of temporal data (waveforms, butterfly plots) and spatial data (topography at time of peak activity in pattern interval). Analysts also provided confidence ratings and rated the typicality of each pattern instance using a 3-point scale.

An initial set of ratings on 100 observations (25 subjects ×4 conditions) was collected. Raters met to discuss results and to calibrate procedures for subsequent ratings. Experts then proceeded to label another 116 ERP observations (4 observations were omitted due to a technical error in the data file). This set of labeled data constituted the “gold standard” for system evaluation.

Interrater reliability for test data was computed for two of the patterns (P100 and N100) using the Spearman-Brown prophecy coefficient [36]. Results are graphed in Table 3 (“*” = moderate reliability, “**” = high reliability).

For both patterns, the highest level of reliability was reflected in the typicality ratings. In addition, reliability was considerably higher for the P100 pattern. Inspection of the data revealed that the low reliability for N100 “presence” judgments was due to a systematic difference in use of categories: one rater consistently rated as “not present” cases where the other rater indicated the pattern was “present” but atypical (“1” on typicality scale).

Accuracy of the autolabeling procedures was defined as the percentage of system labels that matched the gold-standard labels (%Agr; see Table 4). Across the eight patterns, the autolabeling results and expert ratings had an averaged Pearson *r* correlation of +.36. This leads to an effective inter-rater reliability of +.52 as measured by the Spearman-Brown formula. Note that while the %Agr was relatively high for the N100 (0.84), the Spearman-Brown coefficient was considerably lower (0.41), consistent with the lower interrater reliability observed between ERP analysts for this pattern.

## 5. DATA MINING RESULTS

Input to the data mining (“bottom-up”) analyses consisted of 32 metrics for each factor, weighted across each of the 144 labeled observations (total N=4608). Pattern labels for each observation were a combination of the autolabeling results (pattern present versus pattern absent for each factor, for each observation), combined with typicality ratings, as follows. Observations that met the rule criteria (“pattern present” according to autolabeling procedures) and were rated as “typical” (rating > “1”) were assigned to one category label. Observations that *either* failed to meet pattern criteria (“pattern absent”) *or* were rated as atypical (“1” on rating scale), or both, were assigned to a second category. The combined labels were used to capitalize on the high reliability and greater sensitivity of the typicality + presence/absence ratings, as compared with the presence/absence labels by themselves.

For the EM procedures, we set the number of clusters to be 9 (8 patterns + nonpatterns). We then clustered the 144 observations derived from the pattern factors, based on the 32 metrics. As shown in Table 5, the assignment of observations to each of the 9 clusters largely agreed with the results from the top-down (autolabeling) procedures (compare Table 2).

Ideally, each cluster will correspond to a unique ERP pattern. However, as noted above, inaccuracies in either the data summary (tPCA) procedures, or the expert rules, or both, can lead to pattern splitting. Thus, it is not surprising that patterns in our clustering analysis were occasionally assigned to two or more clusters. For instance, the P100 pattern splits into two clusters (clusters 4 and 5), consistent with the autolabeling results (Table 2).

Supervised learning (decision tree) methods were used to derive pattern rules, independently of expert judgments. According to the information gain rankings of the 32 attributes, *TI-max* and *In-mean(ROI)* were most important, consistent with our previous results [22]. These findings validate the use of these two metrics in expert-defined rules. Decision trees revealed the importance of additional spatial metrics, suggesting the need for finer-grained characterization of pattern topographies in our rule definitions. In addition, difference measures (*Pseudo-RareMisses* and *RareMisses-RareHits*) were highly ranked for certain patterns (the N2 and P300, resp.), suggesting that functional metrics may be useful for classification of certain target patterns.

## 6. CONCLUSION

The goal of this study was to define high-level rules and concepts for ERP components in a particular domain (visual word recognition) and to design, evaluate, and optimize an automated data processing and labeling stream that implements these rules and concepts. By combining rule definitions based on expert knowledge (top-down approach) with rule definitions that are generated through data mining (bottom-up approach), we predicted that our system would achieve higher accuracy than a system based on either approach in isolation. Results suggest that the combination of top-down and bottom-up methods is indeed synergistic: while domain knowledge was used effectively to constrain the number of clusters in the data mining, decision tree classifiers revealed the importance of additional metrics, including multiple measures of topography and, for certain patterns, functional metrics that correspond to experiment manipulations.

Ongoing work is focused on the following goals:

- refinement of procedures for expert labeling of patterns in the “raw” (untransformed) ERP data;
- testing of alternative data summary and autolabeling methods;
- modification of rules and concepts, based on integration of bottom-up and top-down classification methods.

### 6.1. Alternative data summary procedures

In the present study, we applied temporal PCA (tPCA) to decompose ERP data into discrete patterns that are input to our automated component classification and labeling process. PCA is a useful approach because it is automated, is data-driven, and has been validated and optimized for decomposition of event-related potentials [21]. At the same time, as illustrated here, PCA is prone to misallocation of variance across the latent factors. Further, differences in the time course of patterns across subjects and experiment conditions are a particular problem for tPCA methods: latency “jitter” can lead to mischaracterization of patterns [7].

For this reason, we are currently testing alternative approaches to ERP component analysis. One approach involves application of sequential (temporo-spatial) PCA. Temporo-spatial PCA is a refinement and extension of temporal PCA (see [12, 19] for details). The factor scores from the temporal PCA, which quantify the extent to which their respective latent factors are present in the ERP data, undergo a spatial PCA. The spatial PCA further decomposes the factor scores into a second tier of latent factors that capture correlations between channels across subjects and conditions. The latent factors from the two decompositions are then combined to yield a finer decomposition of the patterns of variance, that are present in the ERP data.

#### 6.1.1. Windowed analysis of ERPs

The second approach is to adopt the traditional methods of parsing ERP data into discrete temporal “windows” for analysis. By focusing on temporal windows corresponding to known ERP patterns, the algorithms we developed for extracting statistics from the tPCA factors can be extended to the raw ERP, with some modification. While the raw ERP is more complex, with overlapping temporo-spatial patterns, the autolabeling process applied to raw ERPs would correspond directly to the expert “gold standard” labeling procedure. Furthermore, it would not be subject to one weakness of tPCA, namely, that the time courses of the factor loadings are invariant across subjects and conditions.

#### 6.1.2. Microstate analysis

We are also evaluating the use of microstate analysis, an approach to ERP pattern segmentation that was introduced by Lehmann and Skrandies [37]. Microstate analysis is a data parsing technique that partitions the ERP into windows based upon characteristics of its evolving topography. Consecutive time slices, whose topographies are similar under a metric, such as global map similarity, are grouped together into a single microstate. This microstate in turn corresponds to a distinct distribution of neuronal activity. Microstate analysis may hold promise for separating ERP components that have minimal temporal overlap. Moreover, this method has been implemented as a fully automated process (see [38] for downloadable software and [39, 40] for discussion of automated segmentation using microstate analysis).

### 6.2. Development of neural electromagnetic ontologies (NEMO)

In previous work [22] we have described progress on the design of a domain ontology mining framework and its application to EEG data and patterns. This represents a first step in the development of Neural ElectroMagnetic Ontologies (NEMO). The tools that are developed for the NEMO project can be used to support data management and pattern analysis within individual research labs. Beyond this goal, ontology-based data sharing can support collaborative research that would advance the state of the art in EM brain imaging, by allowing for large-scale metaanalysis and high-level integration of patterns across experiments and imaging modalities. Given that researchers currently use different concepts to describe temporal and spatial data, ontology development will require us to develop a common framework to support spatial and temporal references.

A practical goal for the NEMO project is to build a merged ERP-ERF ontology for the reading and language domain. This accomplishment would demonstrate the utility of ontology-based integration of averaged EEG and MEG measures, and make strong contributions to the advancement of multimodal neuroinformatics. To accomplish this goal, we have developed concurrent strategies for representation of ERP and ERF data in sensor space and in source (anatomical) space. To link to these ontology databases and to support integration of EM measures with results from other neuroimaging techniques, we are working to extend our pattern classification process to brain-based coordinate systems, through application of source analysis to dense-array EEG and whole-head MEG datasets.

## Figures and Tables

**Table 1:** Spatial and temporal concepts used to define the eight target patterns. Regions of interest (ROIs) are defined in Appendix A.

Pattern	Window	ROI
P100	60–150	occipital
N100	151–230	occipital
N2	231–300	post-temporal
P1r	250–400	parietal
N3	250–400	left anterior
MFN	250–450	frontal
N4	350–550	parietal
P300	401–700	parietal

**Table 2:** Percentage of ERP observations for each factor that matched expert-defined rule criteria.

	% Observations meeting pattern criteria							
Factor	P100	N100	N2	N3	P1r	MFN	N4	N3
Fac#01	—	—	—	—	—	—	—	—
Fac#02	—	—	—	—	—	**36.81**	**9.72**	59.72
Fac#03	—	**82.64**	—	—	—	—	—	—
Fac#04	**82.64**	—	—	—	—	—	—	—
Fac#05	—	—	—	—	—	—	—	—
Fac#06	—	—	—	—	—	—	—	—
Fac#07	—	—	69.44	**42.36**	**64.58**	22.92	—	—
Fac#08	34.72	—	—	—	—	—	—	—
Fac#09	—	—	—	—	—	—	—	**56.94**
Fac#10	—	51.39	**51.39**	—	—	—	—	—
Fac#11	—	—	—	47.92	25.69	34.03	35.42	—
Fac#12	—	—	—	—	—	—	—	—
Fac#13	—	—	—	59.03	62.50	40.97	—	—
Fac#14	—	—	—	—	—	—	—	—
Fac#15	—	—	—	—	—	—	—	9.72

**Table 3:** Interrater reliability (Spearman-Brown *r*).

	*Presence*	*Confidence*	*Typicality*
P100	.51*	.41*	.72**
N100	−.04	.35*	.45*

**Table 4:** Comparison of autolabeling with expert labels.

Pattern	Person *r*	Spearman-Brwon	%Agr
P100	0.60	0.75	0.90
N100	0.26	0.41	0.84
N2	0.12	0.21	0.53
N3	0.41	0.58	0.63
P1r	0.47	0.64	0.76
MFN	0.33	0.49	0.40
N4	0.37	0.54	0.81
P3	0.30	0.46	0.64

**Table 5:** EM clustering results (NP: nonpatterns).

	**0**	**1**	**2**	**3**	**4**	**5**	**6**	**7**	**8**
P100	0	0	0	0	**60**	**49**	0	0	0
N100	1	0	0	0	0	0	7	30	**77**
N2	**104**	0	0	0	17	0	0	3	8
N3	5	0	0	0	4	2	2	**40**	1
P1r	11	0	14	0	14	6	5	**51**	0
MFN	0	0	0	**56**	0	9	0	0	0
N4	0	0	0	**15**	0	1	0	0	0
P3	0	**113**	0	2	0	0	0	0	0
NP	26	28	22	197	39	16	**33**	64	20

**Table:** 

	**Metric**	**Description**
**Function**	Pseudo-known	Difference in mean intensity over ROI at time of peak latency (Nonwords-Words)
	RareMisses-RareHits	Difference in mean intensity over ROI at time of peak latency (RareMisses-RareHits)
	RareHits-Known	Difference in mean intensity over ROI at time of peak latency (RareHits-Known)
	Pseudo-RareMisses	Difference in mean intensity over ROI at time of peak latency (Nonwords-RareMisses)

**Intensity**	IN-max	Maximum intensity (in microvolts) at time of peak latency
	IN-max to Baseline	Maximum intensity (in microvolts) at time of peak latency with respect to intensity at TI-begin
	IN-min	Maximum intensity (in microvolts) at time of peak latency
	IN-min to Baseline	Maximum intensity (in microvolts) at time of peak latency with respect to intensity at TI-begin
	SP-max	Channel associated with maximum intensity, IN-max
	SP-max ROI	Channel group (ROI) containing SP-max
	SP-min	Channel associated with manimum intensity, IN-min
	SP-min ROI	Channel group (ROI) containing SP-min

**Space**	IN-mean ROI	Mean intensity (in microvolts) at time of peak latency for a specified channel group
	IN-LOCC	Mean intensity (in microvolts) at time of peak latency for left occipital channel group
	IN-ROCC	Mean intensity (in microvolts) at time of peak latency for right occipital channel group
	IN-LPAR	Mean intensity (in microvolts) at time of peak latency for left parietal channel group
	IN-RPAR	Mean intensity (in microvolts) at time of peak latency for right parietal channel group
	IN-LPTEM	Mean intensity (in microvolts) at time of peak latency for left posterior temporal channel group
	IN-RPTEM	Mean intensity (in microvolts) at time of peak latency for right posterior temporal channel group
	IN-LATEM	Mean intensity (in microvolts) at time of peak latency for left anterior temporal channel group
	IN-RATEM	Mean intensity (in microvolts) at time of peak latency for right anterior temporal channel group
	IN-LORB	Mean intensity (in microvolts) at time of peak latency for left orbital channel group
	IN-RORB	Mean intensity (in microvolts) at time of peak latency for right orbital channel group
	IN-LFRON	Mean intensity (in microvolts) at time of peak latency for left frontal channel group
	IN-RFRON	Mean intensity (in microvolts) at time of peak latency for right frontal channel group
	SP-cor	Correlation between factor topography and topography of target pattern

**Time**	TI-max	Latency (in milliseconds) of maximum or minimum amplitude
	TI-begin	Onset (in milliseconds) of waveform excurstion containing peak intensity
	TI-end	Conclusion (in milliseconds) of waveform excurstion containing peak intensity
	TI-duration	Duration (in milliseconds) of pattern, equal to TI-begin minus TI-end

## APPENDICES

### B. ERP PATTERN RULES HYPOTHESIZED FOR VISUAL WORD RECOGNITION

Rule #1 (pattern PT_1_ = P100)
*Let ROI = occipital (average of left and right occipital).* For any n, FA_n_ = PT_1_ *iff*

- 60 ms < TI-max (FA_n_) ≤150 ms AND
- |IN-mean(ROI) |≥.4 mV AND
- IN-mean(ROI) > 0 .

Rule #2 (pattern PT_2_ = N100)
*Let ROI = occipital (average of left and right occipital).* For any n, FA_n_ = PT_2_ *iff*

- 151 ms < TI-max (FA_n_) ≤229 ms AND
- |IN-mean(ROI)|≥.4 mV AND
- IN-mean(ROI) < 0.

Rule #3 (pattern PT_3_ = N2)
*Let ROI = occipital-temporal (average of occipital, posterior temporal).* For any n, FA_n_ = PT_3_ *iff*

- 230 ms < TI-max (FA_n_) ≤300 ms AND
- |IN-mean(ROI)|≥.4 mV AND
- IN-mean(ROI) < 0.

Rule #4 (pattern PT_4_ = N3)
*Let ROI = left anterior temporal.* For any n, FA_n_ = PT_4_ *iff*

- 250 ms < TI-max (FA_n_) ≤400 ms AND
- |IN-mean(ROI)|≥.4 mV AND
- IN-mean(ROI) < 0.

Rule #5 (pattern PT_5_ = P1r)
*Let ROI = parietal temporal (average of left parietal, right parietal)* For any n, FA_n_ = PT_5_ *iff*

- 250 ms ≥ TI-max (FA_n_) ≤ 400 ms AND
- |IN-mean(ROI)|≥.4 mV AND
- IN-mean(ROI) > 0.

Rule #6 (pattern PT_6_ = MFN)
*Let ROI = frontocentral (average of left frontocentral, right frontocentral)* For any n, FA_n_ = PT_6_ *iff*

- 250 ms < TI-max (FA_n_) ≤450 ms AND
- |IN-mean(ROI)|≥.4 mV AND
- IN-mean(ROI) < 0.

Rule #7 (pattern PT_7_ = N4)
*Let ROI = parietal temporal (average of left parietal, right parietal)* For any n, FA_n_ = PT_7_ *iff*

- 350 ms < TI-max (FA_n_) ≤550 ms AND
- |IN-mean(ROI)|≥.4 mV AND
- IN-mean(ROI) < 0.

Rule #8 (pattern PT_8_ = P300)
*Let ROI = parietal temporal (average of left parietal, right parietal)* For any n, FA_n_ = PT_8_ *iff*

- 401 ms ≥ TI-max (FA_n_) ≤700 ms AND
- |IN-mean(ROI)|≥.4 mV AND
- IN-mean(ROI) > 0.

### C. STATISTICAL METRICS

For statistical metrics see Table 6.

## References

[1] Klimesch W (1996). Memory processes, brain oscillations and EEG synchronization. *International Journal of Psychophysiology*.

[2] Jung T-P, Makeig S, Westerfield M, Townsend J, Courchesne E, Sejnowski TJ (2001). Analysis and visualization of single-trial event-related potentials. *Human Brain Mapping*.

[3] Fabiani M, Gratton G, Coles MGH, Cacioppo J, Tassinary L, Berntson G (2000). Event-related brain poten-tials: methods, theory, and applications. *Handbook of Psychophysiology*.

[4] Proverbio AM, Zani A, Zani A, Proverbio AM (2002). Electromagnetic manifestations of mind and brain. *The Cognitive Electrophysiology of Mind and Brain*.

[5] Gasser T, Schuller JC, Gasser US (2005). Correction of muscle artefacts in the EEG power spectrum. *Clinical Neurophysiology*.

[6] Hauk O, Davis MH, Ford M, Pulvermüller F, Marslen-Wilson WD (2006). The time course of visual word recognition as revealed by linear regression analysis of ERP data. *NeuroImage*.

[7] Spencer K, Handy T (2005). Averaging, detection, and classification of single-trials ERPs. *Event-Related Potentials: A Methods Handbook*.

[8] Donchin E, Heffley E, Otto D (1978). Multivariate analysis of event-related potential data: a tutorial review. *Multidisciplinary Perspectives in Event-Related Brain Potential Research*.

[9] Picton TW, Bentin S, Berg P (2000). Guidelines for using human event-related potentials to study cognition: recording standards and publication criteria. *Psychophysiology*.

[10] Nunez PL (1981). *Electric Fields of the Brain: The Neurophysics of EEG*.

[11] Gratton G, Coles MGH, Donchin E (1989). A procedure for using multi-electrode information in the analysis of components of the
event-related potential: Vector filter. *Psychophysiology*.

[12] Spencer KM, Dien J, Donchin E (1999). A componential analysis of the ERP elicited by novel events using a dense electrode array. *Psychophysiology*.

[13] Handy T, Handy T (2005). Basic principles of ERP quantification. *Event-Related Potentials: A Methods Handbook*.

[14] Nobre AC, McCarthy G (1994). Language-related ERPs: scalp distributions and modulation by word type and semantic priming. *Journal of Cognitive Neuroscience*.

[15] Dien J, Frishkoff GA, Cerbone A, Tucker DM (2003). Parametric analysis of event-related potentials in semantic comprehension: evidence for parallel brain mechanisms. *Cognitive Brain Research*.

[16] Frishkoff GA (2007). Hemispheric differences in strong versus weak semantic priming: evidence from event-related brain potentials. *Brain and Language*.

[17] Compton PE, Grossenbacher P, Posner MI, Tucker DM (1991). A cognitive-anatomical approach to attention in lexical access. *Journal of Cognitive Neuroscience*.

[18] Huang K, Itoh K, Suwazono S, Nakada T (2004). Electrophysiological correlates of grapheme-phoneme conversion. *Neuroscience Letters*.

[19] Dien J, Frishkoff GA, Handy T (2005). Introduction to principal components analysis of event-related potentials. *Event-Related Potentials: A Methods Handbook*.

[20] Dien J (2004). PCA toolbox (version 1.093).

[21] Dien J (1998). Addressing misallocation of variance in principal components analysis of event-related potentials. *Brain Topography*.

[22] Dou D, Frishkoff G, Rong J, Frank RM, Malony A, Tucker DM Development of NeuroElectroMagnetic Ontologies (NEMO): a framework for mining brainwave ontologies.

[23] Rong J, Dou D, Frishkoff GA, Frank RM, Malony A, Tucker DM A semi-automatic framework for mining ERP patterns.

[24] Rodriguez-Fornells A, Schmitt BM, Kutas M, Münte TF (2002). Electrophysiological estimates of the time course of semantic and phonological encoding during listening and naming. *Neuropsychologia*.

[25] Dempster AP, Laird NM, Rubin DB (1977). Maximum likelihood from incomplete data via the EM algorithm. *Journal of the Royal Statistical Society Series B*.

[26] Weka 3 Data Mining Software in Java. http://www.cs.waikato.ac.nz/ml/weka/.

[27] Quinlan J (1993). *C4.5: Programs for Machine Learning*.

[28] Frishkoff GA, Perfetti C, Westbury C ERP measures of partial semantics knowledge: left temporal indices of skill differences and lexical quality.

[29] Medler DA, Binder JR MCWord: an online orthographic database of the English language. http://www.neuro.mcw.edu/mcword/.

[30] Wilson MD (1988). The MRC psycholin-guistic database: machine readable dictionary. *Behavioural Research Methods, Instruments and Computers*.

[31] Electrical Geodesics (EGI) Eugene, Oregon. http://www.egi.com/.

[32] Dien J (1998). Issues in the application of the average reference: review, critiques, and recommendations. *Behavior Research Methods, Instruments, and Computers*.

[33] Junghöfer M, Elbert T, Tucker DM, Braun C (1999). The polar average reference effect: a bias in estimating the head surface integral in EEG recording. *Clinical Neurophysiology*.

[34] Junghöfer M, Elbert T, Tucker DM, Rockstroh B (2000). Statistical control of artifacts in dense array EEG/MEG studies. *Psychophysiology*.

[35] McCarthy G, Wood CC (1985). Scalp distributions of event-related potentials: an ambiguity associated with analysis of variance models. *Electroencephalography and Clinical Neurophysiology*.

[36] Rosenthal R, Rosnow R (1991). *Essentials of Behavioral Research: Methods and Data Analysis*.

[37] Lehmann D, Skrandies W (1984). Spatial analysis of evoked potentials in man—a review. *Progress in Neurobiology*.

[38] Cartool software. http://brainmapping.unige.ch/Cartool.htm.

[39] Koenig T, Kochi K, Lehmann D (1998). Event-related electric microstates of the brain differ between words with visual and abstract meaning. *Electroencephalography and Clinical Neurophysiology*.

[40] Koenig T, Lehmann D (1996). Microstates in language-related brain potential maps show noun-verb differences. *Brain and Language*.

